# Lectures Illustrative of Certain Local Nervous Affections

**Published:** 1837-04-01

**Authors:** 


LECTURES ILLUSTRATIVE OF CERTAIN LOCAL NERVOUS AFFECTIONS. By Sir *Benjamin C. Brodie*, Bart. F.R.S. Serjeant Surgeon to the King, and Surgeon to St. George's Hospital. 8vo. pp. 88, London, 1837.

THESE Lectures were originally published in the Medical Gazette. They were published as they were delivered, at intervals, to the clinical class of St. George's Hospital. Their author has now collected them, and offered them to the profession in the shape of a distinct work, which will prove more convenient for perusal and for reference, than in their previous diffused and disjointed form.

The reasons which have induced Sir Benjamin Brodie to adopt this step, induce us to imitate it. We think that a complete view and an accurate analysis of these valuable practical lectures will not be unacceptable nor useless to our readers.

It is obvious that there are two distinct methods of studying and describing disease. One consists in reducing symptoms to their essential causes, and in determining the exact nature of the malady. This is the most precise mode. The other looks upon a group of symptoms as a substantive thing, and makes it an object of special study. For this method of investigation to be practised with advantage, the knowledge derived from the former must be presupposed. It seems the easier method ; not requiring the assistance of morbid anatomy, it was the ancient one. Being necessarily imperfect, it gave rise to the errors of the old pathology, and when those errors were detected by morbid anatomy, men naturally fell into the opposite extreme, and disregarded it altogether.

If all complaints which physicians and surgeons have to treat, arose from organic lesions, there would be no necessity for studying symptoms, except as the exponents of those lesions. We must become familiar with them as the language of disease, and that would be sufficient. But as many of the disorders which occur in practice, are not the consequences of appreciable organic alterations, their symptoms come to be not merely symbols of disease, but, for all useful purposes, to constitute the disease itself. This is especially the case when the nervous system is concerned, and its functional disturbances are so numerous, and so important, as to merit the closest observation.

The nervous system of the female sex is more delicate than our's, its susceptibility of impressions more vivid, its perceptions less regulated by constant laws, and its associations with the functions of the mind and of the body more intricate, or at least more intimate. Its sympathies with the condition of the uterine system are so marked, that the ancients, as is well known, denominated the latter an " animal in animale." Can we wonder then, that nervous disorders should prevail, or that those disorders should be closely linked with the varying conditions of the generative system, in women ?

The customs of civilized society, and the restraints of morality and of religion, conspire to assist the operation of the laws of nature. Woman is peculiarly affected by the restrictions which decorum and the interests of the sex itself, impose on the gratification of the passions. But Nature rebels against the violence done to her, and if the uterus is compelled to remain inactive in those years of life when it is best fitted for the performance of its office, it is often at the expense of serious disturbance to the economy.

It is unnecessary to remark, that the class of complaints we are speaking of have lately attracted a large share of attention, and have been made the subject of investigation by several physicians and surgeons. Some have viewed them as a form and species of hysteria, and, have named them " hysterical," in accordance with that view of their nature and origin. Others have affected to look deeper into the malady, and to refer it to a more exact and a more physiological source, the spinal marrow. " Spinal irritation" is the generic name which these gentlemen have affixed to the multifarious varietes of form in which it shews itself.

Perhaps, as we go on, we shall see reason to believe, that sound objections lie against either name, if that name is really meant to embody the theory it seems to do. Perhaps we shall discover that these hysterical affections are occasionally developed, when there is no appreciable derangement of the uterus—and we are pretty confident that observation and reasoning both conspire to display the fallacy of any view, which regards the spinal marrow or the roots of the spinal nerves, as, in all cases, the essential seat of the disorder. It will probably become apparent, that the whole nervous system is at fault, or, that if parts of it are peculiarly affected, it is difficult to discriminate them with sufficient accuracy, to enable us to pronounce that *there* resides the essential mischief.

Let us turn to Sir Benjamin Brodie, and pursue in detail the facts and reasonings that he advances. The work is made up of three lectures. The first treats of the theory of local nervous affections, of the various circumstances under which they exist, and of the principles on which the treatment of them should be conducted—the second exposes the various forms of local hysterical affections—and the third is devoted to the pathology of hysteria, and to the treatment of local hysterical affections.

### I. Theory of Local Nervous Affections—various Circumstances under which they exist—Principles on which the Treatment of them should be conducted.

The natural sensations of a part may be increased, diminished, or perverted, although no appreciable lesion exists in it. Or, the sensations remaining unaltered, the motions of a part may be similarly affected, and a muscle or a set of muscles may be in a state of permanent contraction, of paralysis, or of spasm. The two cases are analogous. In the former, the nerves of sensation are involved; in the latter those of motion.

It appears that each nerve has a point in the brain or spinal marrow, with which it is in special relation. The sensory filaments go from a portion of the body to that point—the motory filaments pass from that point to the said portion of the body. Thus a sort of nervous circle is maintained, and into it every part of the body supplied with sensory and motor nerves, and a corresponding point in the spinal marrow or the brain must enter. This fact, for it seems to be one, has been the groundwork of Dr. Marshall Hall's ingenious theory of the reflex function of the spinal marrow and medulla oblongata.

The nervous affections resulting from this law are very frequent, and therefore highly important. In one shape or other, says Sir Benjamin Brodie, you will meet with them at every turn of your future practice, and a knowledge of them is of the greatest importance, both to the physician and surgeon. Without it, you will be continually mistaking the real seat of a disease: your attention will be directed to a wrong object, and, following the symptoms, you will be in danger of overlooking the cause on which they depend. The investigation, however, is not unattended with difficulty, and it will often require all your professional sagacity and skill to trace the phenomena, which occur in these cases, to their true origin.

To understand disorders so numerous, and occasionally so obscure, it is necessary to analyse the phenomena that they display, and to reduce them as far as possible to their essential modes. They appear to be three :—

1. Irritation at the root of a sensitive or motor nerve, or in its course, is usually manifested at the termination of the nerve most remote from the brain or spinal marrow. This is a law so striking, and, we may add, so familiarly known, that it is unnecessary to insist at any length on it. But a few examples, selected from the many which the experience of our author has brought within his reach, must be interesting, and may be useful. In the following, the irritation operated on the course of the nerve.

*Case* 1. " A man was admitted into St. George's Hospital in the year 1808, complaining of a severe pain in the inside of his knee. The joint was carefully examined, but no marks of disease could be detected in it. In the thigh, however, there was an aneurysm of the femoral artery, of the size of a small orange. This last disease had scarcely attracted the patient's notice. He said that he should be very well if it were not for the pain in the knee, and it was not until some trouble had been taken to explain to him the nature of his case, that he could be made to understand that the tumour was of any importance. Soon after the man's admission, Sir Everard Home (then Mr. Home) applied a ligature round the femoral artery, in the upper part of the thigh. On the instant of the artery being secured, the tumour ceased to pulsate, and the pain in the knee ceased also. Some untoward circumstances occurred, and the patient died about four or five days after the operation was performed. On inspecting the limb after death, the aneurysm was found reduced to one-half of its former size; some branches of the anterior crural nerve, which passed over it, and which must have been kept on the stretch previous to the operation, were found to terminate in the part to which the pain had been referred, on the inside of the knee; and thus the cause of the pain was sufficiently explained. It was, in fact, a nervous pain, existing where there was no disease, in consequence of pressure on the nerves above." 5.

*Case* 2. " A gentleman in the year 1816 began to suffer from a gnawing pain in the left leg, referred to the course of the peroneal nerve from the foot to the knee. The pain by degrees became very severe, occupying at the same time a larger portion of the limb. The limb itself presented no appearance of disease. The patient consulted various surgeons, myself among the number. The disease went by the name of neuralgia, but the cause of it could not be discovered, and the remedies recommended were of no avail. After having lost sight of him for a considerable time, I was again sent for to see him in the year 1824. He was now dying with dropsy of the belly, and anasarca of the lower limbs. On examining the abdomen it was observed, as the fluid which it contained receded under the pressure of the hand, that there was a large solid tumour attached to the left side of the lumbar vertebræ, and extending into the pelvis. It was evident that this tumour must have pressed on the origin of the sciatic nerve, and thus it afforded a sufficient explanation of the pain which for so many years had been referred to some of its branches." 5.

In the preceding cases, the cause of irritation, pressure, was applied to the nerve between its origin and final distribution: the effect of irritation, pain, was referred to parts in which that distribution took place.

We find similar affections of the motory filaments of nerves. In a case of comminuted fracture, the spicula of bone pressing on a nerve will occasion spasmodic contractions of the muscles, and it is well known that, in experiments, irritation of a motor nerve gives rise to similar consequences. It would seem, however, that painful affections are more frequent than spasmodic ones, from irritation of nerves in their course: and, that, when pressure operates on a motor nerve, paralysis is more common than convulsion.

We may turn to some cases which exhibit the operation of irritation at the spinal or cerebral origins of nerves. They are common enough. An apoplectic extravasation in one hemisphere of the brain, for example, will occasion loss of sensation and motion in the opposite side of the body, perhaps convulsions on the same side. From this extreme case we descend to others of slighter, yet of an analogous description—to an instance of pain in some particular part of the body—of numbness in one hand—of affections of the special senses. Caries of the dorsal vertebræ, irritating the spinal marrow, produces pains and muscular spasms in the lower limbs—while the same disease of the superior cervical vertebræ produces corresponding symptoms in the upper limbs.

*Case.* A gentleman complained of severe pains referred to one side of the abdomen. After having been fixed for some time in one situation, it attacked another. No disease could be detected in the part apparently affected, and the pains were therefore regarded as nervous. It was observed at the same time that his powers of articulation were affected, and that he spoke in an indistinct and drawling manner. This seemed to indicate that there was some disease in the brain, and the suspicion was confirmed soon afterwards by the occurrence of epileptic fits, from which the patient continued to suffer during the few remaining years of his life.

"I mention this case," observes our author, "because I believe that a particular example will serve to impress the fact, which it illustrates, on your minds better than a mere general observation, and not because there is any thing in it in any way remarkable or singular. You will, indeed, when engaged in practice, find nothing more common than this; that a patient consults you, who labours under some disease in the brain, but in whom a particular symptom, referred perhaps to a distant part of the body, is so severe, or so distressing, that he regards this as the original disease; and it is only after a diligent cross-examination that you are enabled to detect the existence of those other symptoms which serve to explain the real nature of the case."

A striking instance of this description occurred to Dr. James Johnson. A woman came under his care on account of severe vomiting. He was unable to discover any local, or indeed any satisfactory cause for it, and the remedies which he suggested were incapable of allaying it. He sent the woman into St. George's Hospital. The physicians who saw her there, experienced the same difficulty in accounting for the symptom, and met with the same ill success in treating it. The woman died. On dissection, there was found disease, we believe an abscess, in the cerebellum, and no other lesion existed.

We had lately an opportunity of witnessing a case, which in some measure resembled the former. A young gentleman was brought to us, on account of obstinate vomiting, and progressive emaciation. The boy looked dull, and had a disproportionately large head, but complained of no pain in it, and had no affection of any of the senses. He had been under the care of several gentlemen, who had not succeeded in arresting the vomiting. We applied leeches and blisters to the head, and the vomiting ceased and has never returned. We purposely refrained from employing any other active remedies, in order to ascertain with more precision the effects of those we have alluded to. Of course, there is nothing in such cases to surprise us, when we reflect on the sickness of stomach from concussion, or on the phenomena exhibited in hydrocephalus or apoplexy. But errors in diagnosis are not unfrequently prevented by the recollection of a striking fact which forcibly intrudes itself on the attention, when general principles are difficult of application, or, from some cause or other, are not applied.

" In many of these cases," continues Sir B. Brodie, " the cause of irritation seems to operate always on the same part of the sensorium, and there is little or no variety in the local indications by which it is rendered manifest. At other times it has no determined seat: it may affect at first one portion of the brain, to which a certain function belongs, and then it may affect another portion, whose function is entirely different, and the symptoms vary accordingly."

*Case.* " A lady became affected with a spasmodic affection of the sterno-cleido mastoideus muscle, producing what is commonly called a spasmodic wry-neck. This symptom continued unabated for a year, and then suddenly left her; but as the spasm in the muscle ceased, she fell into a state of mental depression amounting to insanity; and in this she continued during the whole of the second year. At the end of this period she recovered of the disordered condition of her mind, and the spasm of the muscle returned, continuing from that period up to the time of my being consulted, three or four years afterwards. I was consulted by another lady, in whom a neuralgic affection of the spine alternated with insanity." 8.

2. Although pain is most commonly manifested in the course of the irritated nerve and at a distance from the brain or cord, it appears that at times the irritation is felt at the other extremity of the nerve or at its origin. Perhaps the instance of pain in the back, when port wine is injected into the tunica vaginalis may not be considered unexceptionable. But the case of headache from the presence of irritating matters in the stomach appears a fair example of this fact. The nervus vagus is irritated at its termination by improper matters in that viscus—a headache is the consequence, resembling in its characters the headache of phrenitis itself—and an emetic is instantly followed by *its* disappearance. Who has not seen cases of this description? It is difficult, we think, to explain them, unless we admit that pain may be referred to the origin of a nerve as well as to its distal termination.

3. In other instances a more tortuous route is pursued by sensation, and the effects of irritation are exhibited in a less simple and intelligible manner.

A. Sensation is felt in the ramifications of one nerve, in consequence of irritation exercised upon another, with which it is directly connected at its origin, or in its course.

When a calculus, says Sir Benjamin, passes along the ureter from the kidney into the bladder, it frequently occasions a severe pain in the testicle of the same side. The most probable explanation of this sympathetic affection of the testicle is as follows: many of the nerves of the testicle derive their origin from the renal plexus, which also supplies the kidney, and which is formed by branches of the great sympathetic nerve. The irritating cause, namely the calculus, operates in the first instance on the nerves of the kidney, through which its influence is transmitted to the renal plexus; and from thence it is, as it were, reflected to the nerves of the testicle.

When the testicle is inflamed, it is not uncommon for the patient to complain of pain on the outside of the hip. The following appears to be the explanation. The nerves which supply the scrotum and the cord have the same origin (the lumbar plexus) as the cutaneous nerves of the outside of the hip. The irritation of the former is thus communicated to the latter, and referred to the parts to which *they* are distributed.

Sir Benjamin relates an interesting case of this description. A gentleman laboured under a scrofulous disease of the hip, producing caries of the bones and suppuration within the joint. The following symptoms existed in addition to those which the same disease usually produces. The smallest motion of the thigh induced an attack of excruciating pain, amounting to agony, attended with violent spasmodic contraction of the muscles which move the thigh. The limb was jerked in a most remarkable manner for several minutes, and the volition of the patient had no control over these distressing and extraordinary movements. After some time, a tumor began to present itself externally on the anterior part of the limb, raising the femoral artery, which lay pulsating on its surface. Combined with the disease of the hip-joint, there were scrofulous tubercles and abscesses of the lungs, and of this last-mentioned disease he died, the attacks of pain and spasm having subsided for six or eight weeks before this event took place.

B. In other instances, the mode of connexion between the source of pain and the spot to which it is referred, is more obscure. A lesion in one part of the body causes pain in another and remote part; and we are unable to explain the occurrence, by any communication between the nerves which are primarily implicated and those in which the sensation is made manifest. The most probable supposition, in cases of the nature we are about to quote, is this: *that* the impression is conveyed to the sensorium by one nerve, and transmitted by it to another nerve. Yet it is often impossible to determine *why*, in a given case, a particular route is taken by sensation. The following facts are related by our author.

A gentleman awoke in the middle of the night, labouring under a severe pain in one foot; at the same time that some other sensations, to which he was not unaccustomed, indicated the existence of an unusual quantity of acid in the stomach. To relieve the latter, he swallowed a large dose of an alkaline medicine. Immediately on the acid in the stomach having been thus neutralised, the pain in the foot left him.

"The late Dr. Wollaston was accustomed to relate the following history:— He ate some ice-cream after dinner, which his stomach seemed to be incapable of digesting. Some time afterwards, when he had left the dinner-table to go to the drawing-room, he found himself lame from a violent pain in one ankle. Suddenly he became sick; the ice-cream was rejected from the stomach; and this was followed by an instantaneous relief of the pain in the foot.

A gentleman consulted me concerning a pain in one instep. The pain was severe, causing lameness, so that he walked with difficulty; but there was neither swelling, nor, except the pain, any mark of inflammation. I prescribed some remedies, which, however, were of no avail. One morning he called on me, still suffering from the pain in the foot, and so lame that he could not get out of his carriage and walk into the house without the assistance of his servant. Now, however, he complained of another symptom: he had a difficulty of making water, and a purulent discharge from the urethra. He had laboured under a stricture of the urethra for many years, and had occasionally used bougies. Of late the stricture had caused more inconvenience than usual; but he had abstained from mentioning it, thinking that it would be better that he should (if possible) be relieved of the pain in the foot before any treatment was adopted on account of the stricture. Under these circumstances I introduced a bougie, which penetrated the stricture and entered the bladder. Immediately on the bougie having been used, the pain in the foot abated; and in less than a quarter of an hour he left the house free from pain, and walking without the slightest difficulty. This happened some years ago, but I have seen the patient at intervals ever since; and, from a most careful observation of his case, he and I are both satisfied that the pain in the foot is connected with the disease in the urethra, and we have never found any thing to relieve it except the introduction of the bougie.

A lady consulted me concerning a pain to which she had been for some time subject, beginning in the left ankle, and extending along the instep towards the little toe, and also into the sole of the foot. The pain was described as being very severe. It was unattended by swelling or redness of the skin, but the foot was tender. She laboured also under internal piles, which protruded when she was at the water-closet, at the same time that she lost from them sometimes a large and sometimes a smaller quantity of blood. On a more particular inquiry, I learned that she was free from pain in the foot in the morning; that the pain attacked her as soon as the first evacuation of the bowels had occasioned a protrusion of the piles! that it was especially induced by an evacuation of hard fæces; and that if she passed a day without any evacuation at all, the pain in the foot never troubled her. Having taken all these facts into consideration, I prescribed for her the daily use of a lavement of cold water; that she should take the Ward's paste *(confectio piperis composita)* three times daily, and some lenitive electuary at bed-time. After having persevered in this plan for the space of six weeks, she called on me again. The piles had now ceased to bleed, and in other respects gave her scarcely any inconvenience. The pain in the foot had entirely left her. She observed that, in proportion as the symptoms produced by the piles had abated, the pain in the foot had abated also." 14.

There are cases (that of traumatic tetanus is one), in which the affection of the nervous system is more extensive and more complete. A wound in an extremity has occasionally given rise to epilepsy, and in many diseases an illustration of the fact is exhibited. The irritation occasioned by dentition will, for instance, prove the cause of convulsions—irritation of the mucous membrane of the alimentary canal in children will do the same thing—uterine disturbance will produce epilepsy. Sir B. Brodie relates the case of an officer, in whose leg a musket-ball lodged. At first it produced no inconvenience, but having shifted its place, it occasioned twitches of the muscles of the limb and general convulsions, as in epilepsy. The ball again changed its situation, and these symptoms disappeared.

From a consideration of the laws which regulate the perception of irritation, we naturally pass to the investigation of the conditions under which its phenomena present themselves.

1. Nervous pains are sometimes described as dull and wearying, at other times, and more frequently, as sharp, darting, or stabbing. They may, in the first instance, be readily distinguished from those occasioned by inflammation: by the absence of throbbing, by their not being increased by pressure: by there being no evident turgescence of the small vessels. But there is more difficulty in the diagnosis afterwards. As the commonest event may prove a source of annoyance to an irritable mind, so will nerves, which have been kept for some time in a state of irritation, transmit every impression that is made on them to the brain, with a disagreeable or painful sensation superadded to it: in other words, the part affected will be tender to the touch. And, more than this: the tenderness may be followed by increased vascularity; by a slight degree of swelling; by actual inflammation; not, indeed, continues Sir Benjamin, very active inflammation, but still sufficiently distinctive. In a gentleman who suffered long from what was considered tic douloureux in the face, the parts at last became swollen from an effusion of serum into the cellular tissue, and so exquisitely tender that they would not bear the slightest touch.

2. Nervous pains and muscular spasms both exhibit a marked disposition to be suspended during sleep. They may for a time prevent it, but sleep, having been obtained, is likely to be sound and uninterrupted for many hours. In persons affected with the wry neck, the muscle which is the seat of spasm becomes and remains relaxed during sleep. Exceptions to this law are rare.

3. Nervous pains are usually intermittent. The intermissions are commonly irregular, the patient being subject to paroxysms varying in intensity, in duration, and in frequency, and separated by indefinite intervals. To such paroxysms the term "spasms" is frequently applied; but our author properly objects to its employment, for spasm means contraction, and should be used only to designate involuntary contractions of the muscles.

But occasionally the intermissions are regular, and the returns of the pain periodical, like ague, with which they have been known to alternate. In such cases, the two diseases depend on the same state of system, and are cured by the same remedy, arsenic or quinine. In other cases, however, the character of the pain, whatever that may be, conveys little satisfactory information with respect to either its cause or treatment. The tic douloureux of the face may depend on a projecting spiculum of bone, on carious or dead bone, on disease of the brain, or merely on disorder of the digestive organs. Yet, under these various circumstances, the symptoms may display no material difference.

4. Though any part of the body may be the seat of these affections, some parts appear more prone to them than others.

"They are met with less frequently in the viscera, which are supplied by the great sympathetic nerves, than in other parts. Nervous pains are more severe, and perhaps, on the whole, more common, in those parts which receive their nerves from the fifth pair, as the face, the eye, the tongue, than in any other individual part. Muscular spasms are common in the muscles of the neck, especially in the sterno-cleido-mastoideus. I am inclined to believe, also, that they occur more frequently in the upper limb than in the lower. It is not uncommon to see one hand and arm in a state of constant tremulous motion, there being no other indication of disease. I have seen several cases in which a muscular spasm of the upper limb has shewn itself in the following manner. The patient experiences no inconvenience from it until he uses the limb; for example, until he sits down to write. Then, when he has gone so far as to have written a few letters, some of the muscles act involuntarily, and jerk the hand in a direction contrary to that which was intended; so that, instead of completing the word which was begun, the pen makes a long scratch on the paper." 21.

A lady had her mouth drawn to one side, and was supposed to have paralysis of the opposite side of the face. But, on examination, Sir Benjamin observed nearly constant twitches of the cheek and eyelids on the side to which the mouth was drawn. He satisfied himself that the case was not one of palsy of the muscles of the opposite side, but of spasm of the muscles of *that* side.

The muscles of the pharynx and œsophagus are extremely liable to be affected. In many of the cases which have been confounded under the general appellation of stricture of the œsophagus, the disease is either a spasmodic or a partial paralytic affection of these parts.

" A lady consulted me concerning symptoms which were ascribed to a stricture of the œsophagus. She was unable to swallow the smallest morsel of solid food, so that she was compelled to subsist entirely on liquids, and even these she swallowed with great difficulty. These symptoms had been coming on for upwards of three years. I introduced a full-sized œsophagus bougie, which entered the stomach without meeting the slightest impediment. From this and other circumstances I was led to conclude that the difficulty of deglutition was merely a symptom of some other disease. The lady's face was bleached, as if she had suffered from repeated attacks of hæmorrhage, and her feet were in some degree œdematous. On inquiry, I found that she had long laboured under internal piles, from which had taken place repeated discharges of blood. To this last disease, then, I directed my chief attention, prescribing a cold lavement to be injected every morning; and at the same time a solution of the sulphate of iron, and sulphate of quinine, to be taken three times daily, by the mouth. When this plan had been persevered in for three weeks the piles were much relieved; they no longer protruded externally; there had been no recurrence of hæmorrhage; her cheeks were less pale; and she swallowed with comparative facility. At the end of six weeks more the piles occasioned very little inconvenience; she had lost no more blood; her general health was much improved; and there was so little difficulty of deglutition, that I had no hesitation in recommending that, after her return to the country, she should swallow a bolus of Ward's paste three times daily, with a view to the cure of the hæmorrhoidal disease." 23.

Whatever the pathologist may think, the public are of opinion that the great object of medical science is the cure or alleviation of disease. Morbid anatomy has been latterly studied with industry and with success, but pathology, in a more extended sense, has been perhaps neglected. The Continental school regards patients as the raw material of musea, and the physician fully imbued with its spirit detects a pleurisy or a hepatitis with precision, traces its phases with scientific accuracy, and, after he has let his patient die, he points with triumph to the confirmation of his—diagnosis. In this country, the common sense of the best practical physicians and surgeons has made them chime in with the public sentiment, and prevented them from neglecting that most necessary part of physic—the treatment of disorders. Sir Benjamin Brodie is of the utilitarian school. He has shewn the student the characters and nature of nervous pains, has explained their laws, insisted on the study of their causes, and now he directs attention to their treatment. " Your patient," he says, " wishes to be cured; he has of course no other reason for consulting you." This is a consideration which seems occasionally to be quite forgotten. It is a reasonable one notwithstanding.

The great use of scientific investigation, is to prevent empiricism in treatment. If we have discriminated the various causes of nervous pains, we apply that knowledge to any case, and ascertain with as much precision as possible, the nature of the exciting cause in it. Such a plan will prevent the blind employment of what are called " nervous remedies," and will lead to the selection of such as are appropriate, and to rational experiments where no obvious remedy presents itself. It is almost as important to determine an incurable as a curable case. In the former instance we avoid doing harm, do not nurture false hopes, and support the reputation of medicine by our accuracy.

The treatment may be local or general.

1. Local treatment can be productive of little benefit, when applied to the part to which the pain is referred—supposing that the actual irritation is elsewhere. This is almost a self-evident proposition, and the conviction of it has occasioned the infrequency of operations for tic-douloureux, &c. Perhaps further reformations in this part of practice are still necessary.—The surgeon should be very cautious, for the diagnosis in some cases is difficult, and time and the effects of remedies are occasionally necessary elements in the data for a correct opinion.

"If the original disease operates immediately on the nerves of the affected part, producing in it pain, or muscular spasm or paralysis, you will have first to consider how far it is within the reach of topical remedies. If a tumor presses on a nerve, or if some foreign body, as a musket ball, or a piece of dead bone, irritates its surface, or is intangled in its substance, perhaps the tumor or the foreign body may be removed by a surgical operation, or the tumor may be reduced by other means. If this cannot be accomplished, or if the nerve itself be altered in structure, either from disease or injury, it will become a matter for consideration, whether the limb should be amputated, or whether the nerve should be divided. It is only under these circumstances that any advantage can be expected to arise from the division of the nerve. In ordinary cases of neuralgia, where the disease on which it depends is in the brain, or in some other distant part of the body, or where it is connected with some derangement of the general health, it is evident that such an operation cannot be recommended on any sound principle, and it need be a matter of no surprise that where it is performed it should so generally fail. Where nothing better can be done, and a cure is not within your reach, a palliative treatment may be productive of some advantage, and you may endeavour to mitigate the patient's sufferings by the use of the local vapour bath, or by the application of the opium, or hemlock, or what is still better, the balladonna plaster." 27.

As a disordered state of the digestive organs is a fruitful source of nervous affections, that should, when present, be corrected. When, as not unfrequently happens, a disposition to gout exists, the colchicum and other proper medicines may be given. When regular intermissions and periodical paroxysms occur, the sulphate of quinine, or of the cinchona, combined with arsenic, relieves them.

"But large doses of these medicines are sometimes required. A respectable medical practitioner consulted me, believing that he laboured under a disease of the spine. He complained of pain, which he referred to the inferior dorsal vertebræ, and which was so severe that he could, as he said, scarcely endure it. On inquiry, I learned that the pain attacked him always at a particular period of the night: that it lasted for a certain number of hours, and that he was free from pain, or nearly so, in the intervals. I recommended that he should take the sulphate of quinine procured at Apothecaries' Hall. He took as much as 15 or 16 grains daily without any decided amendment: but I was so satisfied of the efficacy of the remedy in such a case, that I advised him to increase the dose still further. At last he took half dram of the sulphate of quinine daily, and this effected his cure." 29.

Sir Benjamin concludes the Lecture, or Chapter, by relating some odd cases, which it is worth while to recollect.

A. A gentleman who had long laboured under stricture of the urethra, became affected with periodical retention of urine, which recurred at a certain hour every night. The catheter was required once or even twice. After some time Sir B. Brodie prescribed quinine, and only one more attack was experienced.

B. " A lady about sixty years of age complained of a most distressing sensation of thirst, beginning about ten o'clok in the forenoon, continuing for five hours, and recurring daily. A slight degree of chilliness preceded the attack; and while it lasted, although the sense of thirst was such as to produce absolute misery, there was no perceptible dryness of the mouth and fauces, and the secretion of urine was natural. These symptoms had existed for several weeks. The patient appeared to labour under no other disease: she had, however, begun to lose flesh, and her complexion was sallow. The same symptoms had attacked her four years ago. At that time they continued for six months, leaving her thin and debilitated. I prescribed for her three grains of the sulphate of quinine to be taken three times daily. I have not seen her since; but at the end of four days I received a note to the following effect:—" Mrs. ———, the thirsty lady, has the pleasure to say that she is very much better; and she is much obliged to Mr. Brodie for his advice. She returns to the country to-morrow.' "

C. A lady suffered from a neuralgic affection of the face. Her medical attendant prescribed a preparation of valerian, and the pain in the face subsided; but immediately afterwards she began to experience a pain in one foot. This pain recurred in the early part of every evening. After a short time it was followed by redness of the skin, and tumefaction of the subjacent parts near the bases of the toes. These marks of inflammation continued to increase for some hours, and then subsided, leaving the foot of its natural appearance and free from pain. This went on for several months, when Sir Benjamin saw her, and advised the exhibition of quinine. In three or four days, the symptoms entirely vanished.

D. A lady laboured under an inflammation of her leg. The whole leg was swollen from the toes to the knee, the skin being red, painful and tender. These symptoms had existed for several weeks; the usual remedies had been employed, and no amendment had taken place; yet the inflammation did not proceed farther, and there were no signs of suppuration. At last Sir Benjamin observed that the symptoms varied considerably; that sometimes the redness, pain, and swelling had nearly subsided, and that at other times they were as strongly marked as ever; and that these variations always took place on the alternate days. She was now directed to take the sulphate of quinine. The effect was immediate, and a few days completed the cure.

In those cases in which the cause of the local nervous affection is organic disease of the brain or spinal marrow, an ultimate cure is of course out of the question. But the local symptoms may be mitigated by treatment, that is local pains may be so, for muscular spasms or paralysis are little under the control of art, though sometimes they spontaneously subside. For the local pains, applications of hemlock or belladonna, stimulating liniments combined with laudanum, and even blisters, may be advantageously employed.

### II. Various Forms of Local Hysterical Affections.

The second Lecture is occupied with the examination of the forms of local nervous affections. Amongst these, the most interesting, perhaps, to the surgeon, are the nervous affections of the joints. Simulating the organic lesions of the articulations, they are not unfrequently mistaken for them, and a true knowledge of their characters is indispensable to every practical man. These complaints are observed in all ranks, but particularly in the higher, and in *them* they constitute a large proportion of the affections of the joints. As we have on several former occasions alluded to this form of nervous disorders, it is only necessary at present to advert to the more prominent observations of our author. To him is due the merit of having been the first to direct attention to the subject, certainly of having first offered an accurate account of the complaint.

The hip-joint is frequently the seat of this hysterical disorder, and the following is the graphic delineation of it.

"There is pain in the hip and knee, which is aggravated by pressure and the motion of the limb, and the patient often lies fixed in one position on the bed or sofa. You will say, 'are not these indications of a diseased hip joint?' But observe further. The pain is not in general fixed in any one part: it belongs to the whole limb. The patient winces, and sometimes screams, when you make pressure on the hip, but she does the same if you make pressure on the ilium, or on the side as high as the false ribs, or on the thigh, or even on the leg, as low as the ankle: and every where the morbid sensibility is chiefly in the integuments. If you pinch the skin, lifting it at the same time off the subjacent parts, the patient complains more than when you forcibly squeeze the head of the thigh-bone into the socket of the acetabulum. As her attention is more directed to the examination, so the pain, which she suffers from it, is aggravated; and if her mind be occupied in conversation, she will scarcely complain of that, which would have occasioned torture otherwise. Then, there is no wasting of the *glutæi* muscles, and no flattened appearance of the nates; and the aspect of the patient is different from that which you would expect to find if the bones and cartilages of a joint were in a state of ulceration. Neither are there those peculiar and painful startings of the limb at night, attended often with frightful dreams, which mark the existence of this last disease. The pain will sometimes prevent the patient falling asleep, but, if once asleep, she sleeps soundly for many successive hours; and this state of things may continue for weeks, or months, or even for years, without leading to abscess, or any further ill consequences.—There may be a suspicion of abscess (I have known this in a great number of instances), but the suspicion is never realised. Sometimes there is a general tumefaction of the thigh and nates, the consequence either of a turgid state of the small vessels, or of an effusion into the cellular texture (I suppose of the former, as the parts do not *pit*, or remain indented after pressure); but this is entirely different from the swelling of an abscess. In a few rare instances there is a more defined and circumscribed swelling, but still it is altogether different from that of abscess. There is no perceptible fluctuation, and I can compare it to nothing better than a wheal of urticaria of unusual magnitude. A careful examination will always enable you to distinguish these swellings from abscess. For the satisfaction of others, I have sometimes made a puncture with a grooved needle, or some other convenient instrument, the introduction of which would have detected matter, if matter had existed.

I have said that, in these cases, there is no wasting of the glutæi muscles, and no flattened appearance of the nates. It is, however, not uncommon to find much alteration in the figure of the parts, of another kind; namely a bulging of the pelvis posteriorly, at the same time that it is elevated, on the side of the disease, so as to make an acute, instead of a right angle, with the column of the vertebræ. Of course, under these circumstances, the limb is apparently shortened, and when the patient stands erect, the heel does not come in contact with the ground. A superficial observer may be led to believe that there is an actual dislocation of the hip; and, indeed, it requires a careful examination to enable the surgeon to understand that all this strange distortion is but the result of the predominant action of certain muscles, and of a long-continued indulgence in an unnatural position." 40.

It is scarcely necessary to dwell on the symptoms of this affection of the knee, or of any of the other joints. In all cases we observe, to a greater or a less extent, the following circumstances. 1. The pain is at least as much on the surface, as in the interior of the joint. 2. It is more diffused throughout the limb, than disease of the articulation would account for. 3. It is excited by such gentle manipulations as could hardly disturb the deeper structures, or give rise to pain dependent on disturbance of those structures. 4. It is not produced in a proportionate degree by pressure of the articulating surfaces together, or by those methods of examination which occasion pain when the joint is actually diseased. 5. However excruciating it may appear at one moment, it will not be exhibited the next, if the patient's attention is adroitly drawn off. 6. We may say that in very many cases pain may be found in various parts of the body, provided the patient's attention is ingeniously directed to the part to be examined. 7. There *are* the general marks of the hysterical habit, and there *are not* the usual evidences of serious organic disease.

Our author proceeds to point out some further circumstances, which contribute to the completion of the history of the disorder, and to the assistance of the surgeon in his diagnosis.

A. The patients are, for the most part, not much above the age of puberty.

B. The menstruation is frequently irregular, but sometimes it is not.

C. Those who labour under habitual coldness of the hands, have a weak small pulse, and afford other indications of a feeble circulation, are more liable than others to suffer in this manner; yet occasionally we find these symptoms existing in combination with a florid countenance and a sufficient development of animal heat.

D. In some instances the joint to which the symptoms are referred, and even the whole limb, is affected with a remarkable alternation of heat and cold. Thus in the morning the limb may be cold, and of a pale or purple colour, as if there were scarcely any circulation of blood in it; while towards the afternoon it becomes warm, and in the evening is actually hot to the touch, with the vessels turgid and the skin shining. Sir Benjamin has never known any bad consequences result.

E. The patients may have had hysteria, prior to the occurrence of the local symptoms. The former may recur and the latter be abated or quite pass away.

F. A severe illness inducing great exhaustion, may have preceded the development of these symptoms. Or they may be traced to a moral cause.

A moral agency too may cure them. In many cases we cannot reasonably blame the patient, or lay the symptoms to the account of dissimulation.—But, on the other hand, it must be admitted, *that* females of the strongest minds and most moral sentiments are both least prone to these affections, and most easily cured when they have occurred—*that* in some instances the mind does appear to be strangely warped—and *that* sufferings are exaggerated, if not absolutely feigned. Our judgment must be formed in each case upon its circumstances, but good feeling and reason combine to incline the surgeon to the opinion most favourable to the honour of his patient.

"Although there are none of those painful and involuntary startings of the limbs which occur in combination with caries of the joints, spasmodic actions of the muscles of the limbs are not uncommon in the cases of which I am now speaking. In some instances convulsive motions of the limbs are produced by pinching, or even by lightly touching the integuments. These bear no very distant resemblance to the movements of chorea; and it is worthy of notice, that they do not occur if it can be managed, at the same time, that the attention of the patient should be otherwise directed. I have also known them to take place independently of any manifest exciting cause. In some cases, which have fallen under my observation, the limb was at irregular periods violently agitated, so as almost to throw the patient off her couch." 43.

There is always a sense of weakness in the limb, which, of course is augmented in proportion to the continuance of inaction of the muscles. When the other symptoms have passed away, this may remain; and if the limb has been long in the horizontal posture, the foot, when first put to the ground, assumes almost a purple colour, the consequence of a yet imperfect circulation.

The symptoms for the most part come on gradually, and gradually too, they pass away. But occasionally they vanish all at once, and, as they are not the result of an actual organic lesion, we cannot experience surprise at their doing so. A young lady, for example, was seen by Sir B. Brodie in 1834. She laboured under symptoms simulating disease of the hip-joint. A short time back, Sir Benjamin was informed by Dr. Mortimer, surgeon of Haslar Hospital, that her symptoms continued nearly unaltered for two years, when, one night, on turning herself in bed, she said that she had a feeling as if something had given way in her hip, and from that moment she was well. It is of consequence to remember that such sudden cures may happen, because they will explain the influence of trifling remedies, of quackery, or of religious enthusiasm. Such miracles as those of Hohenlohe are most easily performed on patients of this character.

The spine is not unfrequently the seat of these affections. They are far too often confounded with disease of the bodies of the vertebræ or of the intervertebral cartilages, and this mistake entails on the unhappy patient caustic issues, setons, long confinement in the horizontal posture, the serious remedies in short for a serious disease. This is indeed a cruel blunder. A young lady is confined during the best years of her life to the horizontal posture, tortured by severe remedies, perhaps irretrievable damaged in health, when pure air, gentle exercise, and cheerful occupations might have banished the disorder in the course of a few months.

"In these cases the patient complains of pain and tenderness of the back, to which one or more of the following symptoms may be superadded tending very much to mislead the medical or surgical attendant:—Pains in the limbs, especially in the lower limbs; a sense of constriction of the chest; involuntary spasms of the muscles, sometimes induced by change of position, at other times recurring without any very evident cause; a sense of weakness in the lower limbs, so that they are scarcely capable of supporting the weight of the body; and even actual paralysis; difficulty of voiding the urine. When the patient first complains of pain in the back, it must be acknowledged that there is some difficulty in forming a positive diagnosis; but the difficulty vanishes afterwards, so that none but a superficial observer can have any doubt as to the real nature of the disease. The pain in the back is seldom confined to a single spot, but it extends to different regions of the spine, and it not unfrequently shifts its place from one part to another. The tenderness of the spine is peculiar. The morbid sensibility is chiefly in the skin, and the patient, for the most part, flinches more when the skin is even slightly pinched, than when pressure is made on the vertebræ themselves. The pain is, in the majority of cases, more severe than in those of real vertebral disease; and the spasms of the muscles have a near resemblance to those of chorea. Where there is paralysis, or a tendency to paralysis, it is quite different from what is observed in cases of pressure on the spinal cord or brain; and I may take this opportunity of observing, with respect to hysterical paralysis generally, that it has this peculiarity: *it is not that the muscles are incapable of obeying the act of volition, but that the function of volition is not exercised.* The accuracy of this observation will, if I am not much mistaken, be acknowledged by all those who are at the pains of studying these cases with the attention which they so well deserve; and the importance of it in medical and surgical practice is sufficiently obvious. There are still other circumstances which may assist us in forming our judgment; such as the general aspect of the patient, and her condition in other respects; her time of life; the state of the menstruation; and especially the liability to the more common hysterical affection." 48.

Surgeons occasionally apply a hot sponge, as a means of distinguishing caries of the spine. This is not unlike the old ordeal by fire—there is no getting out of it. If merely touching the skin, gives pain in nervous affections, it can hardly be anticipated that applying a hot sponge to it, will not give pain too. In short, neither reasoning nor experience countenance such a mode of diagnosis.

Retention of urine is well known to be a common hysterical symptom. It is not that the muscles concerned in the act of expelling the urine are incapable of obeying volition, but that volition itself is not exercised.—When this has been the case for a certain length of time, the bladder, constantly distended, becomes really paralysed, and may be affected with chronic inflammation of its mucous membrane. In a case which occurred to Sir B. Brodie, hysterical retention of urine having been long neglected, forty ounces of urine were at last drawn off by the catheter; and, on the post mortem examination, the bladder was found of very large size and of almost a black colour, its muscular fibres much wasted, and its mucous membrane presenting the appearance of a very thin fibre, readily separable from the parts below.

"Females who labour under hysterical retention of urine, if left to themselves, usually recover in the course of a short space of time; sometimes almost suddenly; but if the catheter be employed, their recovery may be protracted for an indefinite period. We may lay it down as a general rule, that in these cases the catheter should not be had recourse to: and the only exceptions to it are in those extreme cases in which actual paralysis has taken place, and the bladder is likely to become diseased, if not artificially relieved." 50.

Hysterical retention of urine affords, perhaps, as good an instance as any of the composite character of these affections. If a girl in a hospital labours under this retention, and if a dresser or house-surgeon can be got to draw off her water regularly, she makes no effort to relieve herself, nay, will rather suffer to a great degree than do so. It is impossible for any one who is practically conversant with cases of this sort, not to be convinced that the patients *like* the species of attention they receive. But if a catheter is secured in the bladder, and the manipulations of the surgeon are no longer necessary, they usually soon get tired of the restraint, and void the urine without the assistance of an instrument. Nothing is more common than such an occurrence in hospitals, and the surgeon must remember that it is the character of many of these nervous symptoms to grow by indulgence, and to nurture, if great caution is not exercised, perverted moral sentiments.

Hysterical aphonia, or loss of voice, is another symptom which may take place suddenly, continue long, and disappear as suddenly as it began. This is not unfrequent in the male sex, especially in persons of the clerical profession, whose voices are occasionally much exerted.

Nor is tympanitis rare in hysterical females. Sir Benjamin conjectures that the majority of cases of ovarian dropsy, supposed to be cured by iodine and other remedies, have been really instances of tympanitis. The diagnosis is obvious, nor is it difficult. On percussion, fluctuation is absent, and extreme resonance is present. The patient floats in the warm bath, if accidentally put into it. An elastic gum tube, passed into the upper part of the rectum, will evacuate, if pressure is made on the abdomen, the greater part of the air, which soon, however, re-accumulates. A stimulating injection, made with the confectio rutæ, will sometimes produce a similar effect.

We can experience no surprise at finding that the breast is occasionally the seat of these hysterical affections. The case has been described by Sir Astley Cooper, in his Observations on the Diseases of the Mamma. The symptoms wear the family likeness. Heightened sensibility, residing not only in the gland but in the skin, and not only in the breast, but in its vicinity, and even at a distance from it—the absence of complaints of pain, if the girl's attention is diverted from the local examination—the want of any tumor in the first instance, and the presence of only slight enlargement or of general fulness, even when the malady has existed long—and, finally the general condition of the individual, are here, as elsewhere, the distinctive characters of a hysterical disorder.

"These cases are to be distinguished from those of a rare kind of irritable tumour of the breast, of which a representation is to be found among the plates annexed to Sir Astley Cooper's work. I conceive that they ought also to be distinguished from those which may occur at any time of life, and in women who have no particular disposition to hysteria. In the cases to which I now allude, the pain and tenderness are much less than in the true hysterical affection of the breast, and it will be almost invariably found that the patient has witnessed the miseries of some friend or acquaintance who has suffered from carcinoma. No part of the body will bear that rigid scrutiny to which the breast is subjected under these circumstances. Close attention will discover in any, even in the most healthy organ, sensations which had been previously overlooked; and constant anxiety on the subject may magnify such sensations into pain. In these last-mentioned cases a strong assurance that no disease exists will make the patient happy, and remove the pain; but no such assurance will be adequate to the cure of a genuine hysterical affection." 54.

Constipation of the bowels is a frequent occurrence in hysterical patients. Sir B. Brodie has known this mistaken for stricture of the rectum. Indeed it is extraordinary what a facility some surgeons possess in discovering stricture of the rectum. Constipation being complained of, a long bougie is introduced into the bowel. It hitches in some of the folds, or is obstructed at its curve on the brim of the pelvis. That is conclusive evidence of the existence of stricture of the rectum, and the patient is subjected to an useless and a worse than useless course (at all events to *him)* of long bougies. To revert to the hysterical constipation we were speaking of: Sir B. Brodie suspects that, in the majority of instances, it is similar in its nature to hysterical retention of urine, that is, the patient does not properly exert volition, unless the accumulation of fæces is excessive.

Hysterical difficulty of deglutition is probably an analogous affection. It is likely to be mistaken for stricture of the œsophagus. Yet it is not that the muscles are in a state of spasm, but that they are not effectually exerted.

Symptoms resembling those of tetanus occasionally present themselves in hysterical individuals. They may assume the form of trismus, or of opisthotonos—and it may be well believed that *such* tetanus is curable, and has been reported cured.

If these local hysterical affections are developed without any local injury, it is, à fortiori, probable that, in persons prone to them, they would be likely to occur when such injury has directed the patient's attention to a part. Now this is an important circumstance to bear in mind, for it will correct some erroneous opinions which have been generally entertained. Symptoms have been constantly attributed to injuries of particular nerves, and operations have been proposed or performed in accordance with such notions, when the source of the disorder was to be sought in the general state of the system. Sir B. Brodie's remarks on this head are valuable.

"A woman (he observes) is bled in the arm. She complains, perhaps, of severe pain at the time; but this subsides, and the wound heals, as under ordinary circumstances. Then she complains of pain again, extending down the forearm to the hand, up the arm to the axilla and shoulder, and even to the side of the neck, and sometimes down the side of the chest also: the extent and degree of pain varying in different cases. You examine the cicatrix, but can discover nothing unusual in it; but the patient flinches when it is touched. She very commonly complains of the surgeon, saying that she was badly bled, or bled with a blunt lancet, or a foul lancet, or that a nerve was pricked which ought not to have been touched; while the real origin of her symptoms may be traced to the peculiar state of her own nervous system. If you investigate the case further, you will always find that she has been liable to various nervous symptoms previously to those which are attributed to her being bled; and when these last disappear, nervous symptoms of some other kind shew themselves.

In another case, the patient has received a blow on the head. In order to avert the consequences which such an injury may be expected to produce, she is bled repeatedly, takes aperient medicines, and is kept on a low diet. When her physical powers are thus reduced, she complains of pain in the head even more than she did in the first instance: but the pain is of a different character, and is usually attended with other symptoms, such as do not belong to inflammation. Thus she has a sense of dizziness, or a feeling as if water was trickling over her head. Then the countenance is blanched, the skin is cool, and the pulse is probably small and quick, and weak. If under these circumstances, the surgeon, mistaking the nature of the case, continues to abstract blood, and to keep the patient on a low diet, all these symptoms become aggravated; other symptoms of a more decidedly hysterical character shew themselves, and no improvement takes place until a more judicious treatment is adopted. In another case, which is of no unfrequent occurrence, a young woman pricks her finger, or perhaps the finger is merely pinched. Soon afterwards, she complains of pain extending from the finger upwards, along the hand and fore-arm. This probably is followed by a convulsive action of the muscles of the arm, or by a continued contraction of the flexor muscles on the anterior part of the arm, so that the fore-arm is kept permanently bent; at least while the patient is awake, for the spasm is generally relaxed during sleep." 57.

The symptoms, which, in these patients, sometimes follow a local injury, occasionally proceed to an extent which could hardly be anticipated.

*Case.* A young lady, eleven or twelve years of age, pricked the fore-finger of her left hand with the point of a pair of scissars. This was immediately followed by pain in the course of the median nerve, and on the following day the fore-arm was fixed, by muscular contraction, at a right angle with the arm. After a few days, all the muscles of the hand and fore-arm were affected with violent spasms, producing strange convulsive movements of the hand and fore-arm. These were attended with sickness and vomiting, so that for two days, whatever was received into the stomach was immediately rejected from it. By degrees the other limbs became affected in the same manner, and it was impossible for the patient to walk, or even to stand. Sometimes the diaphragm was affected so as almost to threaten suffocation. At other times the jaw was closed by a contraction of the masseter muscle, or she lay in a state of opisthotonos. Occasionally there was a violent pain in the head, which was described as having the same character as that of the finger which had been pricked. These symptoms continued to alternate with each other for some time, when recovery took place.

"I have seen several cases of a singular affection of the hand and wrist, which manifestly belongs to the class of cases of which we are now treating. It occurs in females who have a disposition to hysteria, especially in those who have suffered from mental anxiety and over-exertion, and is usually, but not constantly, referred to a sprain, or some other slight accident. The patient complains of pain in the back of the hand and wrist, trifling at first, but gradually becoming more severe. In many instances, after some time has elapsed, there is a diffused swelling of the soft parts, extending a short distance up the lower extremity of the fore-arm; and downwards as low as the fingers. This swelling is not attended with redness of the skin; and having lasted for a few weeks, it subsides, while the pain remains, constant in its character, aggravated by every motion of the limb, and always more severe in proportion as the patient's attention is in a greater degree directed to it. To prevent the motion, which she so much dreads, the patient keeps her hand in one position, and the consequence is that the joints become comparatively stiff, the hand at the same time having a very characteristic appearance, the skin being smooth and shining, and appearing to adhere more closely than is usual to the parts beneath. This state of things may continue for three months, for six months, or even for one or two years; the symptoms then gradually subsiding, without leading to any further ill consequences. The result, however, is not always so fortunate. I attended a lady who laboured under the symptoms which I have just described, with the late Dr. Luke. She left London on a visit to the continent, without any amendment having taken place. I saw her again after the lapse of four or five years: the muscles of the fore-arm were at this time wasted and paralytic; the whole hand was shrivelled and useless; the fingers permanently contracted towards the palm of the hand; the nails thin and scabrous." 60.

Sir Benjamin concludes the lecture, by mentioning some rare and rather whimsical cases, which have fallen under his own observation.

1. He was consulted on the case of a young lady, who was liable to fits of incessant sneezing, attended with a most abundant flow of watery fluid from the nostrils, in which there was no perceptible disease. She had also occasionally nervous cough, globus hystericus, or ordinary hysteria.

2. A married lady, aged 37, had similar fits of sneezing and watery discharge from the nostrils. These symptoms attacked her once a week, and in each attack she sneezed not less than a hundred times. She had also disagreable nervous sensations in the face and palate. At the end of three years from the commencement of the disorder, the fits of sneezing occurred only once in a month, but she complained of an aching pain, and a sense of pulsation in the roof of the mouth, the teeth, and tongue, occurring chiefly in the night and then severe.

3. An unmarried lady, aged 32, had distressing paroxysms of dyspnœa, attended with great general agitation and excitement. The singular feature of the case was this:—There was a particular spot near the ensiform cartilage, which she believed to be in some way or another connected with her complaint. Nothing could be discovered in this part different from what is usual, by the most strict examination; but the pressure of the finger on it never failed to induce one of the paroxysms before alluded to. When these were most severe, they were attended with an abundant flow of limpid urine. They had existed more or less for ten or twelve years, and originally supervened on the exhaustion occasioned by an attack of typhus fever.

4. A young married lady, who was liable to ordinary attacks of hysteria, complained of a tender spot on the anterior part of the abdomen, a little below the ensiform cartilage. The slightest pressure of the finger on it caused excessive pain, and was followed by violent agitation of the whole person, bearing a more near resemblance to the convulsive motions of *chorea* than to any thing else, and continuing for several minutes.

Here we are compelled to pause. The conciseness of our author's style precludes much condensation—the value of his facts forbids many omissions—and, insensibly, we have exceeded the limits permitted to us. One lecture remains. A valuable one, because it inculcates the principles of treatment, but too long to enable us to pursue it now. We will return to it in our next number, and conclude our account of this excellent practical volume.

